# A Rare Case Report of a Paratesticular Fibrous Pseudotumor Mimicking a Hydrocele

**DOI:** 10.7759/cureus.1565

**Published:** 2017-08-14

**Authors:** Srinivasan Dharanya, Chellappa Vijayakumar, Sundaramurthi Sudharsanan, Sadasivan Jagdish

**Affiliations:** 1 Otorhinolaryngology, Jawaharlal Institute of Postgraduate Medical Education and Research (JIPMER), Puducherry, India.; 2 Surgery, Jawaharlal Institute of Postgraduate Medical Education and Research (JIPMER), Puducherry, India.

**Keywords:** testicular tumor, orchiectomy, fibrous pseudotumor, paratesticular tumor, hydrocele

## Abstract

Tumors arising in the spermatic cord are very rare. The common benign tumors of the spermatic cord include adenomatoid tumor, lipoma, neurofibroma, and leiomyoma. We report a rare case of fibrous pseudotumor of the spermatic cord presenting clinically as a hydrocele. A 28-year-old male presented with the complaint of swelling in the left scrotum, which gradually progressed in size over four years. Clinically, the swelling was soft and fluctuant. The left testis was not separately made out and a diagnosis of left hydrocele was made. On scrotal exploration, a large 12 x 7 cm fleshy mass was seen in the left hemiscrotum with the left testis adherent to the upper pole of the mass. The mass was dissected out from the testis, and the histology showed features of fibrous pseudotumor of the spermatic cord. Fibrous pseudotumor of the spermatic cord is a very rare entity and can pose a diagnostic challenge. Preoperative scrotal ultrasound and intraoperative frozen section assessment can prevent unnecessary orchiectomies in young patients with paratesticular fibrous pseudotumors.

## Introduction

Paratesticular fibrous pseudotumors are rare lesions involving the testicular tunics and the paratesticular soft tissues. The etiology is not known, though they are thought to be a reactive condition following trauma or infection. They typically present as painless multinodular scrotal masses that are usually firm to hard in consistency. Clinically, they may mimic a malignancy, but they are composed of dense fibrous tissue with interspersed bland fibroblasts and myofibroblasts and mixed inflammatory cells. Herein, we describe a rare case of a diffuse fibrous pseudotumor that presented clinically as a soft, fluctuant scrotal swelling mimicking a hydrocele. This case report highlights the importance of clinical examination and preoperative ultrasonography in patients presenting clinically with a hydrocele, especially in high volume government hospitals in developing countries.

## Case presentation

A 28-year-old male presented to the surgery outpatient department with the complaint of swelling in the scrotum for a period of four years. The swelling gradually progressed in size over four years and was not associated with pain or fever. There was no history of prior surgery or trauma to the scrotum. On examination, a 12 x 7 cm non-tender swelling was noted in the left hemiscrotum, and it was soft and fluctuant. The left testis was not separately palpable and we could get above the swelling. The testis on the right side was normal. A diagnosis of left hydrocele was made, and a left-sided eversion of sac under local anesthesia was planned.

**Figure 1:**
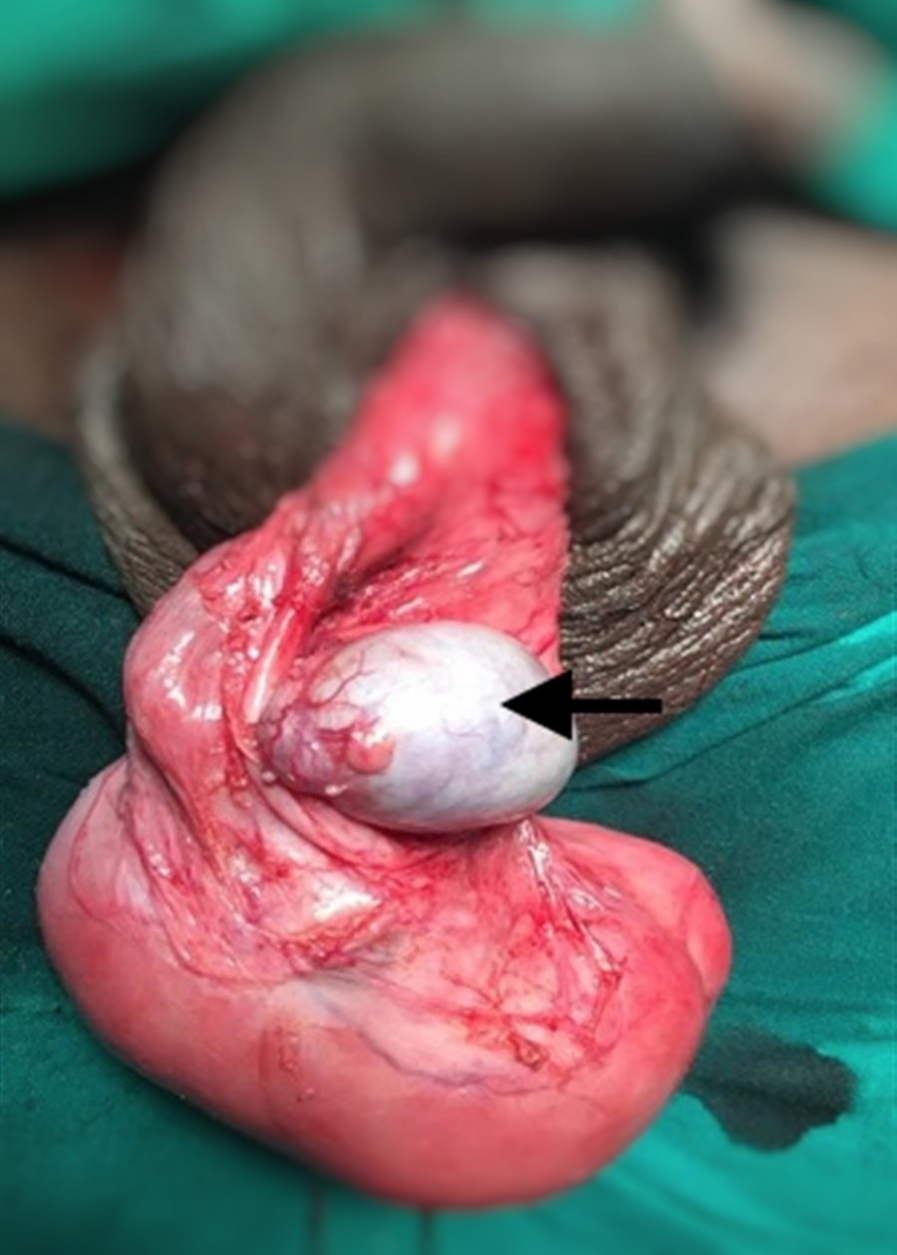
Intraoperative picture of the left scrotal mass with the testis (arrow) adherent to the upper pole of the mass.

On scrotal exploration under the spermatic cord block, a large 12 x 7 cm pink fleshy mass was seen in the left hemiscrotum with the left testis densely adherent to the upper pole of the mass (Figure 1).

**Figure 2:**
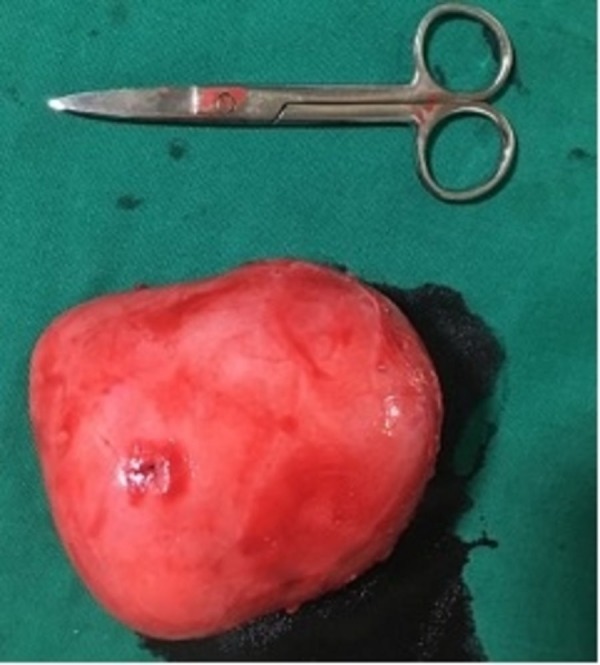
Resected gross specimen measuring approximately 12 x 7 cm.

**Figure 3:**
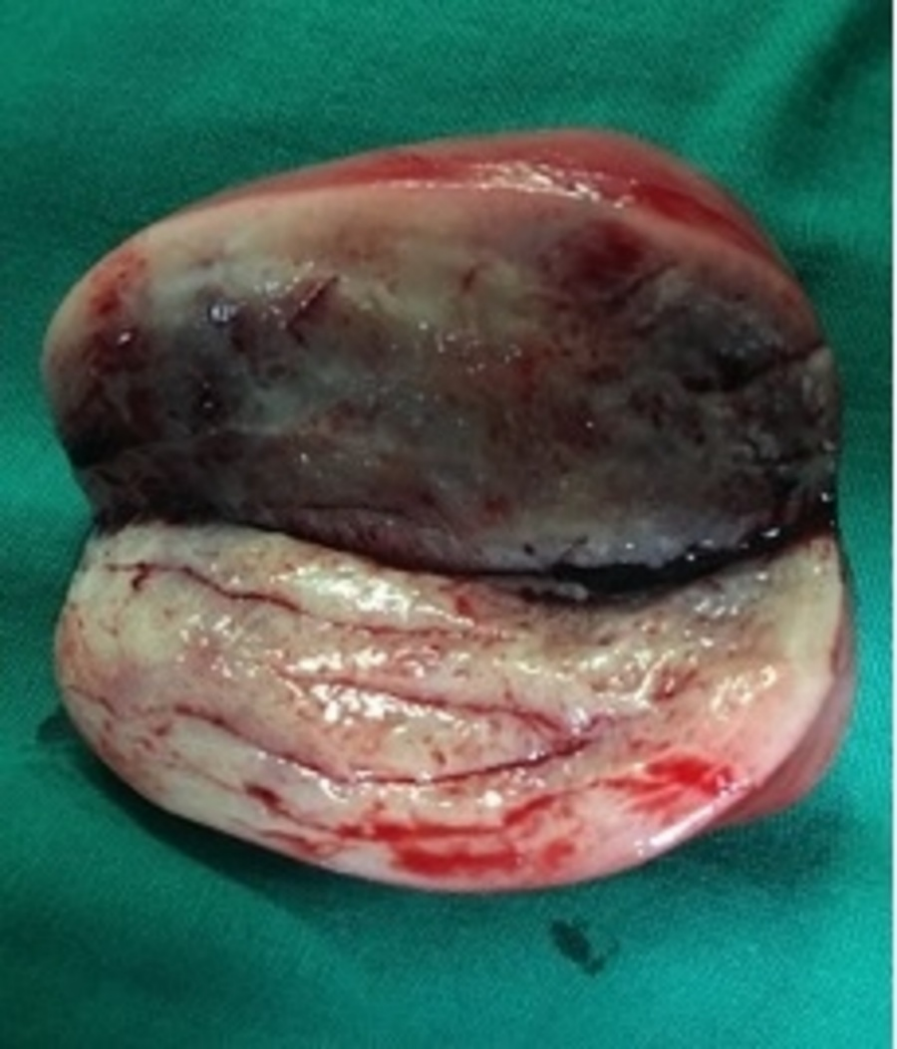
Cut section of the uniform, firm rubbery mass.

The mass was dissected from the testis and removed in toto (Figures 2-3) and was sent for histopathological examination.

**Figure 4:**
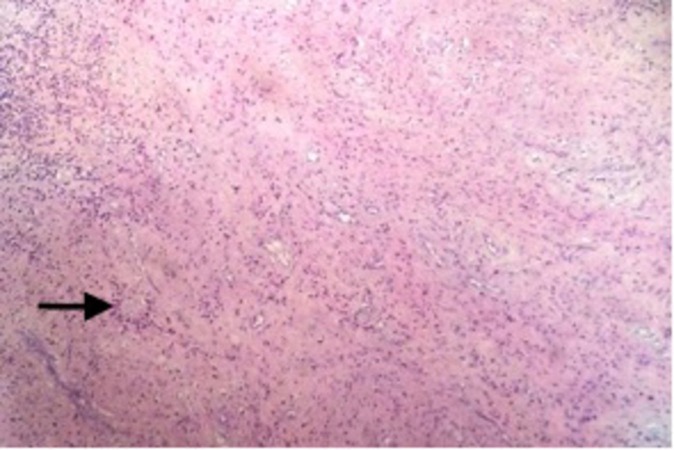
(Hematoxylin & Eosin, 200x ) The section shows tumor composed of spindle-shaped cells with blood vessels admixed with chronic inflammation (marked by arrow).

The patient made an uneventful recovery. Histology of the mass showed a tumor composed of spindle-shaped cells with blood vessels admixed with chronic inflammation (Figure 4).

**Figure 5:**
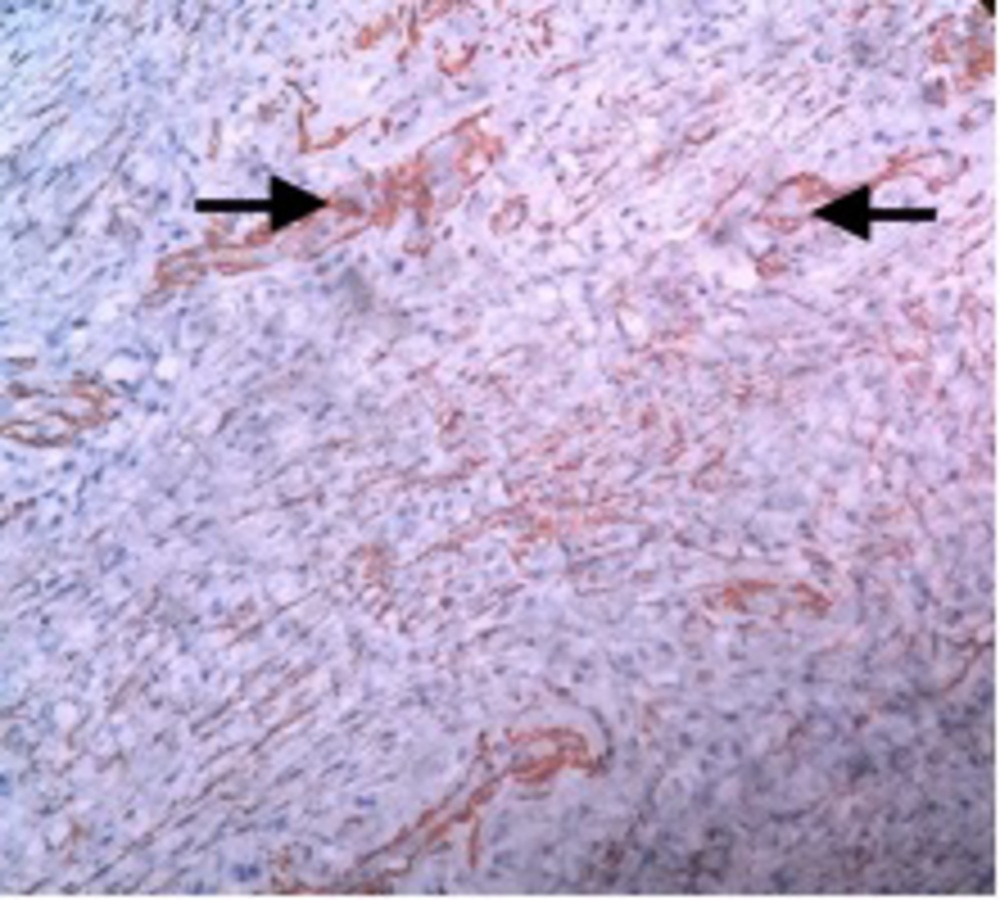
Immunohistochemistry with SMA shows focal positivity (arrow). SMA - smooth muscle actin.

**Figure 6:**
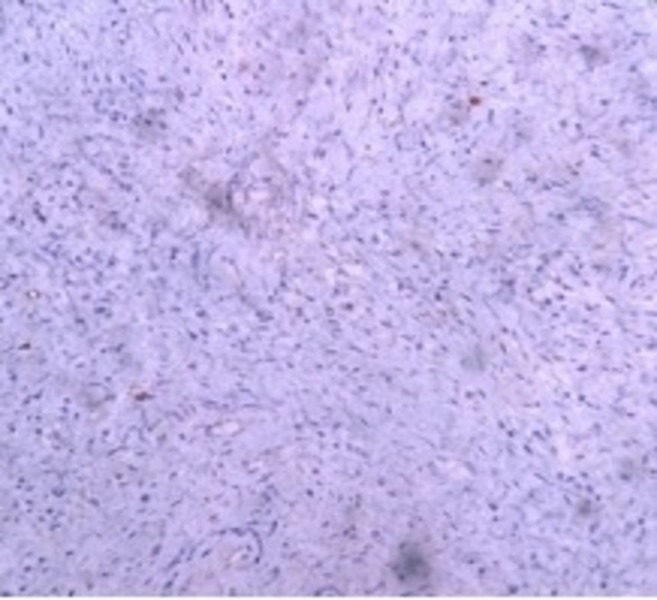
Immunohistochemistry with Ki67 showing low proliferation of the tumor cells (poor staining for Ki67).

Immunohistochemistry with smooth muscle actin (SMA) showed focal positivity (Figure 5) and with Ki67 showed low proliferation of the tumor cells (Figure 6).

Based on the histology and immunohistochemistry features, the tumor was reported as a fibrous pseudotumor of the spermatic cord.

## Discussion

Paratesticular fibromatous lesions were recognized by Sir Astley Cooper in 1830. Numerous terminologies are used to describe this condition based on the variation in the gross and microscopic appearance, differences in the opinion on cell of origin, their location, and the benign or malignant nature of these lesions. The terminologies include chronic proliferative periorchitis, inflammatory pseudotumor, nodular and diffuse fibrous proliferation, proliferative funiculitis, fibromatous periorchitis, fibroma, benign fibrous paratesticular tumor, fibrous mesothelioma, and pseudofibromatous periorchitis [1]. The term paratesticular fibrous pesudotumor is commonly used and is thought to be a reactive, non-neoplastic condition.

Paratesticular fibrous pseudotumors were first reported in detail by Balloch in 1904. Paratesticular tumors are very rare and fibrous pseudotumors constitute about 6% of these paratesticular tumors [2]. These tumors originate from the fibroblasts or myofibroblasts. The exact pathogenesis of these lesions remains unclear though infection, trauma, and autoimmune mechanisms are described in literature [3]. They commonly present in the third decade of life, though they can occur at any age. Fibrous pseudotumors commonly involve the tunica vaginalis in 75% of the cases followed by the epididymis in 10% of the cases. Tumors involving the spermatic cord are extremely rare [4].

They usually present as a unilateral painless mass and are more common on the left side. The size ranges from 0.5 to 8 cm, though tumors as large as 25 cm have been reported [5]. Most patients present with multiple stony nodules, but rarely present as a single diffuse mass as in our case. The consistency is hard, mimicking a testicular tumor, but in our case it was soft and fluctuant. A history of scrotal trauma, surgery, or infection may be present in one-third of the cases. The lesion is associated with hydrocele in 30 to 50% of the cases [6].

Grossly, the fibrous pseudotumors are well-circumscribed, firm, uniform gray-white or yellow lesions. Cystic changes, areas of necrosis, hemorrhage, and calcifications are rare. On microscopy, these lesions are characterized by the presence of myofibroblast and fibroblast spindle cells in a collagenous or myxoid matrix. A compact proliferation of spindle cells arranged in storiform or fascicular growth pattern are present in a few cases. Inflammatory infiltrate comprising of plasma cells, lymphocytes, and occasional eosinophils can be seen. Immunohistochemical staining is positive for vimentin, smooth muscle-specific actin, and common muscle actin and is negative for S-100, keratin, and desmin [7].

Paratesticular fibrous pseudotumors may be associated with IgG4-related diseases like retroperitoneal fibrosis, sclerosing pancreatitis and cholangitis, Riedel thyroiditis, and sclerosing sialadenitis. An elevated level of intralesional IgG4-expressing plasma cells is seen in some of these tumors [8]. However, our case did not have signs of these IgG4-related diseases.

Scrotal ultrasonography is the initial investigation modality in patients with paratesticular tumors. Ultrasound differentiates between an intratesticular or extratesticular lesion and if the lesion is solid or cystic. Fibrous pseudotumors are usually uniformly hypoechoic but can be hyperechoic depending on the amount of collagen and the presence of calcification. Magnetic resonance imaging (MRI) can give additional information when an ultrasound is inconclusive and can provide better tissue characterization and tumor delineation. These lesions typically appear as low intensity lesions in T1 and T2 weighted images due of the presence of fibrosis [9]. The disadvantage of imaging by ultrasound or MRI is that they cannot differentiate between benign or malignant lesions.

Surgical excision is the treatment of choice for paratesticular fibrous pseudotumors. Radical orchiectomy may be needed in cases where the lesion is diffuse and encasing the testis. Intraoperative frozen section biopsy can prevent orchiectomy in a young patient and is recommended when the testis is involved by the tumor [10]. The prognosis for benign fibrous paratesticular tumor is excellent and recurrence after complete surgical excision has not been reported in literature.

## Conclusions

Fibrous pseudotumors are a rare cause of scrotal swellings and they can mimic testicular tumors. Preoperative diagnosis is challenging and a high index of suspicion is needed especially in young patients with hard scrotal swellings. This patient presented with a soft fluctuant mass, which was probably due to the high fat content of the tumor in this case, the finding being termed pseudofluctuation. In developing countries where the surgery outpatient attendance is too high in government hospitals, scrotal ultrasound is not routinely done in a clinically diagnosed case of hydrocele. Preoperative scrotal ultrasound and intraoperative frozen section assessment can prevent unnecessary orchiectomies in young patients with paratesticular fibrous pseudotumors.

## References

[1] Jones MA, Young RH, Scully RE (1997). Benign fibromatous tumors of the testis and paratesticular region: a report of 9 cases with a proposed classification of fibromatous tumors and tumor-like lesions. Am J Surg Pathol.

[2] Bhandari A, Elder JS, MacLennan GT (2008). Fibrous pseudotumor of the tunica vaginalis. J Urol.

[3] Tunuguntla H, Mishra A, Jorda M (2011). Inflammatory myofibroblastic tumor of the epididymis: case report and review of the literature. Urology.

[4] Woodward PJ, Schwab CM, Sesterhenn IA (2003). From the archives of the AFIP: extratesticular scrotal masses—radiologic-pathologic correlation. Radiographics.

[5] Elem B, Patil PS, Lambert TK (1989). Giant fibrous pseudotumor of the testicular tunics in association with schistosoma haematobium infection. J Urol.

[6] Parker PM, Pugliese JM, Allen RC Jr. (2006). Benign fibrous pseudotumor of tunica vaginalis testis. Urology.

[7] Khallouk A, Ahallal Y, Tazi E (2011). Benign paratesticular fibrous pseudotumor with malignant clinical features. Rev Urol.

[8] Bösmüller H, von Weyhern CH, Adam P (2011). Paratesticular fibrous pseudotumor-an IgG4-related disorder?. Virchows Arch.

[9] Muglia V, Tucci S, Elias J (2002). Magnetic resonance imaging of scrotal diseases: when it makes the difference. Urology.

[10] Subik MK, Gordetsky J, Yao JL (2012). Frozen section assessment in testicular and paratesticular lesions suspicious for malignancy: its role in preventing unnecessary orchiectomy. Hum Pathol.

